# LH supplementation of ovarian stimulation protocols influences follicular fluid steroid composition contributing to the improvement of ovarian response in poor responder women

**DOI:** 10.1038/s41598-020-69325-z

**Published:** 2020-07-31

**Authors:** S. Marchiani, L. Tamburrino, F. Benini, M. Pallecchi, C. Bignozzi, A. Conforti, C. Alviggi, L. Vignozzi, G. Danza, S. Pellegrini, E. Baldi

**Affiliations:** 1 Azienda Ospedaliero Universitaria Careggi, 50134 Florence, Italy; 2 Department of Experimental and Clinical Medicine, University of Florence, 50139 Florence, Italy; 3 Centro Procreazione Assistita Demetra, 50141 Florence, Italy; 4 Department of Experimental, Clinical, and Biomedical Sciences, University of Florence, 50139 Florence, Italy; 5 Department of Neuroscience, Reproductive Sciences and Dentistry, School of Medicine, University of Naples Federico II, 80138 Naples, Italy

**Keywords:** Infertility, Mass spectrometry, Steroid hormones, Reproductive techniques

## Abstract

In this prospective study, we evaluated the steroid levels in 111 follicular fluids (FF) collected from 13 women stimulated with FSH monotherapy and 205 FF collected from 28 women stimulated with FSH + LH because of a previous history of hypo-responsiveness to FSH. Steroid levels were measured by HPLC/MS–MS and related to ovarian stimulation protocol, oocyte maturity, fertilization and quality of blastocysts, after individually tracking the fate of all retrieved oocytes. 17-Hydroxy-Progesterone, Androstenedione, Estradiol and Estrone were significantly higher in the FSH + LH protocol. Progesterone, 17-Hydroxy-Progesterone and Estradiol were more expressed in FF yielding a mature oocyte (p < 0.01) in the FSH + LH protocol. FF Progesterone concentration was correlated with the rate of normal fertilization in the FSH protocol. None of the FF steroids measured were associated with blastocyst quality and achievement of pregnancy. Our results indicate that LH supplementation in hypo-responsive women modifies ovarian steroid production, mimicking physiological production better and likely contributing to an improved ovarian response. Employing a correct methodological procedure to evaluate the relationship between FF steroid hormones and assisted reproduction outcomes, our study reveals that some steroids in single follicles may be helpful in predicting oocyte maturity and fertilization.

## Introduction

Infertility is a global problem estimated to affect about 80 million couples in the world. Infertile couples may rely on assisted reproduction techniques (ARTs) which include IVF (in vitro fertilization) and ICSI (intracytoplasmic sperm injection) to achieve a pregnancy. These treatments not only aim at solving fertility problems but also offer a great opportunity to study human reproductive physiology more in depth, presenting the chance to benefit from biological fluids otherwise impossible to obtain. For instance, human follicular fluid (FF) samples provide a crucial microenvironment involved in oocyte development. Their composition depends largely on secretions of granulosa and thecal cells and is influenced by hormonal, paracrine and autocrine signals^1^. In ARTs, all mature retrieved oocytes are usually fertilized but only a part of them develops into an embryo which results in a successful implantation. Clarifying which factors in FF regulate the development and acquisition of oocyte competence is a major goal of current research. FF is easily available as it is aspirated together with the oocyte during ovum pick up and is suitable for evaluation of the constituents involved in oocyte maturation and acquisition of fertilization competence.

Steroids are the main components of FF. They influence oocyte development both directly and indirectly (by acting on somatic cells within the follicle). During ART, ovarian stimulation protocols modify FF steroid composition and their levels regarding natural cycles, likely impacting the resulting oocyte quality^2,3^. There is evidence also that different stimulation protocols may affect steroid levels^4,5^ as well as other components^6^ of FF. In particular, studies comparing FF steroid hormones in women stimulated with FSH or FSH + LH, according to clinical practice^7–9^, show inconsistent results^5,10–12^.

Several studies evaluated FF steroid levels in relation to oocyte maturity^13^ or ART outcomes^4,5,14–16^, but results are conflicting. The controversy may depend on the fact that some of these studies utilized pooled FF^5,17^, others considered only the individual FF retrieved from the first or the dominant follicle^14,15^ or evaluated steroid levels only in those follicles leading to an embryo transfer^4^. Although the recent meta-analysis by Nagy et al.^16^ reported a positive association between progesterone FF levels and fertilization rate, the Authors concluded that the results should be interpreted with caution, because the included studies are very heterogeneous regarding couple inclusion/exclusion criteria, women stimulation protocols, and methods of evaluation of the steroids. The method used to evaluate steroids is important. Immunoassays, which have been used in most studies^4,5,14,18,19^ are affected by cross-reactivity and show poor specificity and sensitivity. Conversely, gas chromatography/mass spectrometry and high-performance liquid chromatography/tandem mass spectrometry (HPLC–MS/MS) analysis using stable isotope labeled steroids are characterized by high specificity and sensitivity^20–22^. HPLC–MS/MS is currently considered the gold standard method for steroid hormone measurement in clinical research^23^. The HPLC–MS/MS method has been used in few studies to evaluate the association between FF steroid levels and reproductive outcomes^4,15,17^. In particular, the study by Kushnir et al.^4^, evaluating the relationship between FF steroid hormone levels and live birth, did not observe any association (with the exception of pregnenolone which was lower in cycles with low birth rate). In the study by Walters et al.^15^, none of the FF steroids were associated with pregnancy or other ART outcomes except for estradiol which was negatively related to the number of blastocysts. It should be considered that the study by Kushnir included only 14 stimulated women for a total of 22 FF, whereas that of Walters et al.^15^, although studying a larger cohort (77 women), evaluated FF steroid levels only in the dominant follicle of each woman.

The two principal aims of the present study were: (1) to evaluate whether the composition and the levels of FF steroids were influenced by ovarian stimulation protocol and (2) to relate FF steroid levels to oocyte maturation and in vitro fertilization outcomes. The level of six steroid hormones [Progesterone (P), 17-Hydroxy-Progesterone(17-OH-P), Androstenedione (A), Testosterone (T), Estrone (E1) and Estradiol (E2)] were measured using the HPLC–MS/MS method in each retrieved follicle collected from 41 women, 13 stimulated with FSH and 28 with FSH + LH, according to clinical practice.

## Materials and methods

### Study design and participants

41 couples undergoing ICSI cycles at the Demetra ART Center of Florence (Italy) from November 2016 to August 2018 were enrolled (Table 1 reports the baseline characteristics of the participating couples).

**Table 1:** Baseline characteristics of the participating couples in the two groups of ovarian stimulation (FSH and FSH + LH) and in the total cohort.

Parameters	FSH group	FSH + LH group	p	Total cohort
Number of couples	13	28		41
Female age (years, median value [IQR])	34.0 [31.5–36.0]	36.0 [33.0–39.0]	**< 0.0001**	35.0 [33.0–38.0]
Male factor	4/13 (30.8%)	10/28 (35.7%)	0.80	14/41 (34.1%)
Unexplained	1/13 (7.6%)	12/28 (42.9%)	**0.01**	13/41 (31.7%)
Number of follicles (average number of follicles/woman)	121 (9.3)	230 (8.2)	0.35	351
Number of MII oocytes	96/121 (79%)	163/230 (71%)	0.12	259/351 (74%)
Number of fertilized oocytes	74/96 (77%)	119/163 (73%)	0.47	193/259 (75%)
Number of cleaved embryos	36/74 (49%)	44/119 (37%)	0.11	80/193 (41%)
Number of high-quality blastocysts	21/36 (58%)	14/44 (32%)	**0.02**	35/80 (44%)
Number of intermediate quality blastocysts	12/36 (33%)	17/44 (39%)	0.62	29/80 (36%)
Number of low-quality blastocysts	3/36 (9%)	13/44 (29%)	**0.02**	16/80 (20%)
Number of pregnancies/Number of transferred (fresh or frozen) blastocysts	9/22 (41%)	7/32 (22%)	0.10	16/54 (30%)

p column indicates the statistical significance between the two groups of ovarian stimulation. In bold statistically significant differences.

Women were included in the study according to the following criteria.

- Age ≤ 43 years
- Normal body mass index (BMI) from 18 to 26 kg/m^2^
- Basal Follicle stimulating hormone (FSH) < 10mUI/ml; Antimüllerian hormone (AMH) > 1.5 ng/ml; Antral Follicle Count (AFC) > 6
- Tubal and/or male factor or idiopathic infertility

The exclusion criteria were:

- Polycystic ovary syndrome (PCOS) according to Rotterdam criteria^24^
- Ovarian surgery
- Pelvic endometriosis^25^
- Poor ovarian response according ESHRE 2010^26^

A team of gynecologists and embryologists coordinated all the steps of the study, ensuring that oocyte pick up procedures, culture protocols and embryo assessment were standardized.

### Ovarian stimulation protocols

All the patients were treated according to the short standard ovarian stimulation protocol consisting of gonadotropin stimulation from day 2 of the cycle, combined with a flexible antagonist protocol (cetrorelix 0.25 mg/day Cetrotide, MERCK SERONO, Germany or ganirelix 0.25 mg, Orgalutran, MSD, Italy). In 13 cases follicle stimulation was performed with r–FSH monotherapy (Gonal F, MERKSERONO, Germany or Puregon, MSD, Italy). In 28 women with normal ovarian reserve parameters, but previous unexpected suboptimal or hypo-response to ovarian stimulation (Poseidon Group 1–2^7–9^), r-LH 75–150 IU (Luveris, MERCK SERONO, Germany) was added from day 1 of stimulation. This subgroup of women belongs to the novel stratification proposed by POSEIDON (Patient-Oriented Strategies Encompassing IndividualizeD Oocyte Number) group^7,8,27^ and is characterized by low IVF prognosis in terms of cumulative live birth rate compared to the former group treated with r-FSH monotherapy^28^. As proposed by POSEIDON Group^9,29^ we opted for FSH and LH co-treatment in this study group. The starting dose of r-FSH ranged from 150 to 225 IU, according to age and response to previous ovarian stimulation. The starting dosage was then modified according to patient response assessed by serum estradiol levels and ultrasound evaluation at 2-day intervals, until at least two follicles reached or exceeded a mean diameter of 17 mm. Finally, oocyte maturation was induced by injection of 5,000 IU of u-hCG (Gonasi, IBSA FARMACEUTICI, Italy) or 250 µg r-hCG (Ovitrelle, MERCK SERONO, Germany). Gonadotropin stimulation and GnRH-antagonist was continued until the day of hCG triggering. Each individual oocyte and the individual corresponding FF were collected separately about 35 h later using a sonographically guided puncture of each follicle, under sedation and local anesthesia. Luteal support was given to all patients, administered as intravaginal micronized progesterone (Progeffik, 200 mg three times daily, EFFIK, Italy), from the day after oocyte pick up until 12 days after embryo transfer, when serum βhCG was measured. In the event of positive βhCG levels, clinical pregnancy was verified by ultrasonographic visualization of the gestational sac about 15 days later and, consequently, classified as pregnant or non-pregnant.

**Figure 1:**
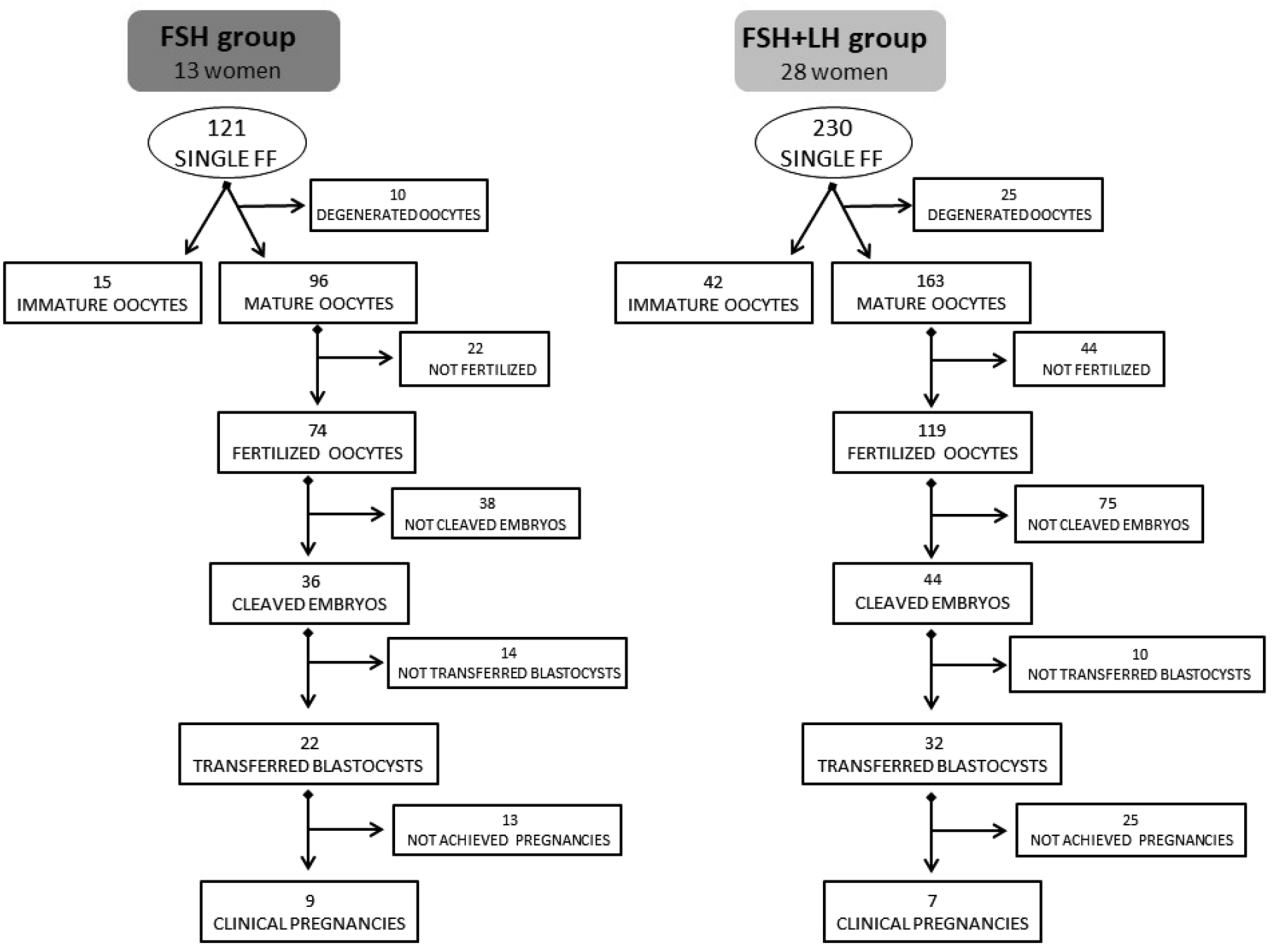
Flowchart of the fate of the single collected oocytes in the two groups of ovarian stimulation. Analyses were performed on 111 FF in FSH group and on 205 FF in FSH + LH group.

A schematic representation of sample distribution and numbers of mature/immature and fertilized oocytes, embryo development, embryo transfer and clinical pregnancy in the two groups of ovarian stimulation is shown in Fig. 1.

### Collection and handling of follicular fluids

Oocyte pick-up was performed under vaginal ultrasound guidance, each follicle was aspirated separately and placed in a different dish.

The ovum aspiration was performed using double lumen needles (COOK, Australia) which were washed with flushing medium to avoid FF contamination between the different aspirated follicles. Each FF collected and the corresponding retrieved oocyte were recorded with a code so that the resulting embryo could be clearly identified with the same code. The volume and the appearance of each FF sample was recorded. Only aspirates of follicles which were uncontaminated with blood and contained an oocyte were included in the analysis. Each collected FF was centrifuged, divided into aliquots of 200 µl, stored in sterile tubes at − 20 °C and individually analyzed for steroid content. After excluding blood contaminated FF and those retrieved from oocyte-lacking follicles, a total of 351 FF and their corresponding oocytes were included in the study. Of these, 35 were subsequently excluded because the oocytes were degenerated (Fig. 1). Each oocyte was individually followed during the entire IVF cycle to track its development after fertilization.

### Oocyte maturity, ICSI procedure and outcomes

All collected oocytes were observed under microscopy and classified into two groups according to their maturity degree: mature (oocytes in metaphase II) and immature (oocytes in metaphase I or germinal vesicles). For ICSI, Nikon Eclipse TE2000-S microscope equipped with Narishige IM-9B Microinjector (NIKON, Japan) was used. After 17 ± 1 h from microinjection, oocytes were observed under microscopy for the presence of two pronuclei to assess the occurrence of correct fertilization and, consequently, classified as fertilized and non-fertilized oocytes. Cultures of each embryo were extended to blastocyst stage.

Continuous Single Culture Complete medium (IRVINE SCIENTIFIC, Santa Ana, CA) was used for embryo culture. After 44 ± 1 and 68 ± 1 h, pace of division, degree of fragmentation, size and symmetry of the blastomeres were evaluated by microscopy. Blastocysts were incubated in a MIRI incubator (ESCO MEDICAL GROUP, Denmark) at 37 °C, 6% CO_2_ and 5% O_2_ and were scored according to the Gardner criteria^30^. The degree of blastocyst development is represented by a numerical value between 1 and 6 and the overall quality of the trophectoderm and inner cell mass indicated by a letter grade between A and C. Surplus transferable embryos were cryopreserved. Based on the criteria described above, embryos were divided into three groups: high quality (HQ), intermediate quality (IQ) and low quality (LQ) according to Supplemental Table 1.

After 5 days post oocyte retrieval, a single blastocyst, chosen on the basis of the morphologic criteria described above, was transferred into the uterus of each woman. The remaining blastocysts were cryopreserved. In 14 women for whom the fresh cycle did not result in a clinical pregnancy, a single frozen blastocyst was subsequently transferred.

### High performance liquid chromatography coupled with tandem mass spectrometry (HPLC–MS/MS) for evaluation of steroid hormones

Steroid levels were evaluated in 316 FF deriving from mature and immature oocytes (Fig. 1). P,17-OH-P, A, T, E2 and E1 and their respective internal standards (Progesterone-2H3, 17α-Hydroxyprogesterone-13C3, Androstenedione-13C3, Testosterone-2H3, Estradiol-2H3 and Estrone-2H4) were purchased from SIGMA-ALDRICH (USA) and stocked at 1,000 µg/ml in methanol. Water and Methanol were ULC-MS grade by BIOSOLVE BV while Formic Acid, Ammonium Formate and Ammonium Fluoride were provided by SIGMA-ALDRICH (USA).

P, 17-OH-P, A and T were analysed in positive ion mode while E2 and E1 in negative mode in two different runs. The MS conditions and the acquired transitions for each steroid are reported in Supplemental Table 2. Briefly, the internal standards in 100 µl of methanol were added to follicular fluids (5 µl), samples were centrifuged, the supernatant diluted to 1000 µl with water and 10 µl were injected.

Cleanup and concentration was performed in isocratic conditions (Water/Methanol 95/5) on a Phenomenex Luna 5 µm C18 20 × 2 mm column whereas the analytical separation was carried out in gradient mode using a Phenomenex Luna 3 µm C18 50 × 2 mm for the positive Ion mode acquisition (androgens analysis), or the Phenomenex Luna 3 µm PFP(2) 50 × 2 mm for the negative ion mode acquisition (estrogens analysis). Both columns were maintained at 40 °C.

For the analysis in positive ion mode, the mobile phase consisted in water and methanol containing 10 mM of formic acid and 5 mM ammonium acetate buffer. The separations in negative ion mode were performed using water added with 0.2 mM ammonium fluoride and methanol.

### Statistical analysis

**Figure 2:**
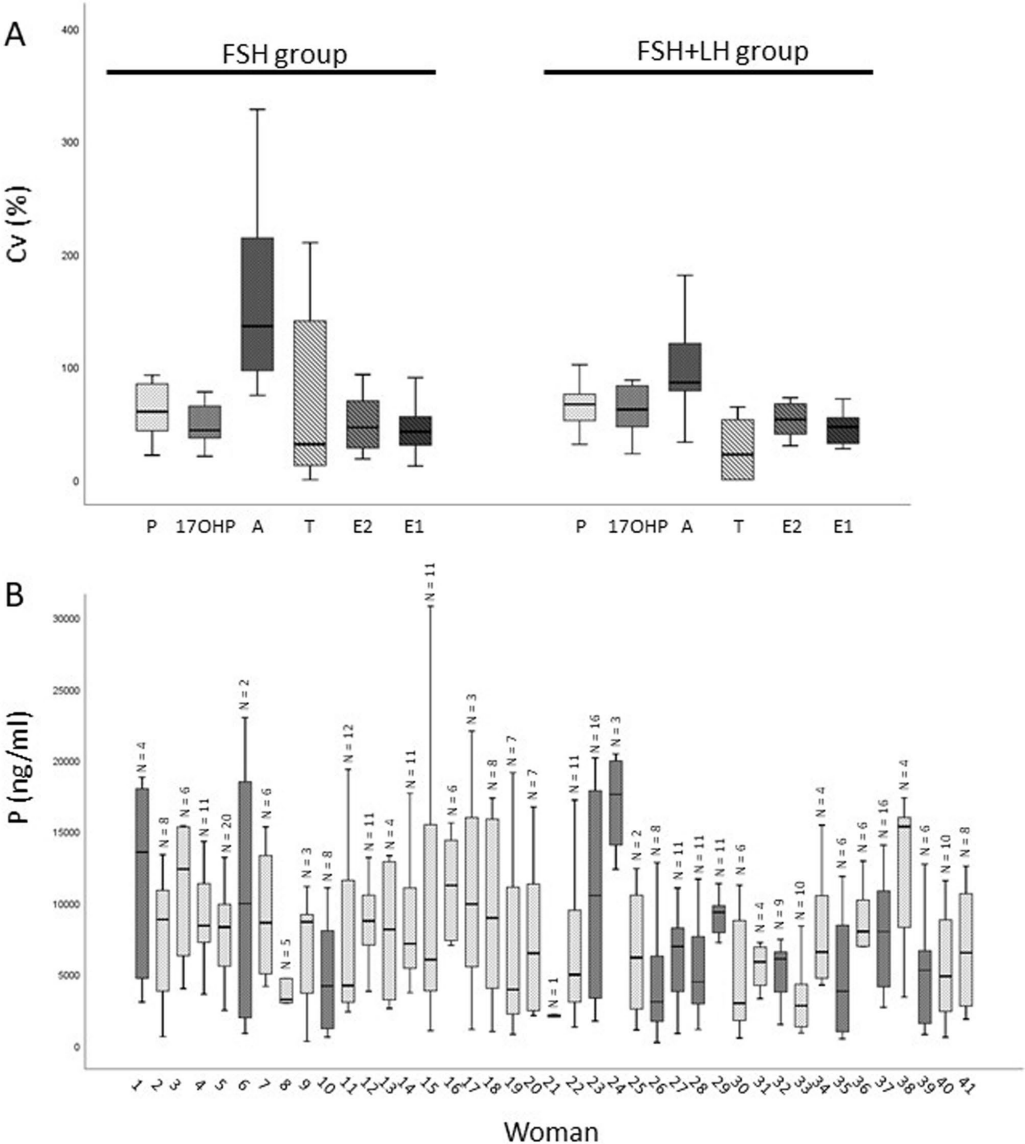
(**A**) Boxplot of median (IQR) coefficients of variation (CV, %) of steroid hormone FF levels in the two groups of ovarian stimulation. Progesterone (P), 17α-Hydroxyprogesterone (17-OH-P), Androstenedione (A), Testosterone (T), Estradiol (E2) and Estrone (E1). (**B**) Median values (IQR) of P levels in the 41 recruited women as an example of inter- and intra-subject variability of steroid FF levels. Light grey boxes: FSH group women (n = 13); dark grey boxes: FSH + LH group women (n = 28). N reports the number of analyzed FF in each woman.

Data were analyzed with SPSS (STATISTICAL PACKAGE FOR THE SOCIAL SCIENCES, USA), version 25.0 for Windows. All the continuous variables displayed a non-normal distribution (as evaluated by Kolmogorov–Smirnov test) and therefore were expressed as median (interquartile range-IQR) values. Because the variability of measurements among individual follicles of a patient was comparable to that among the different women (Fig. 2), each FF (and the corresponding oocyte) was analyzed as a single event. Considering that statistically significant differences were observed between the two ovarian stimulation protocols for some outcomes (Table 1), all the statistical analyses were conducted in the two groups (FSH and FSH + LH) separately. For the categorical variables, Chi-square test was used to evaluate differences between the two groups. Inter-subject variability was expressed as coefficient of variation (CV) = (SD/mean of the determinations) × 100. Correlations among steroid hormone levels were assessed using Spearman’s method. Mann–Whitney U test was used for comparisons between the following groups: mature vs. immature oocytes, fertilized vs. non-fertilized oocytes, high vs. intermediate vs. low embryo quality, pregnant vs. non-pregnant. Logistic binary regressions were applied for multivariate analyses to adjust data for male factor^31^ and female age^32^, two relevant factors impacting ART success. Female factor was not considered because only women with tubal factor (which is overcome by in vitro fertilization) were included. Male factor is defined, according to the WHO criteria^33^, as the presence of at least one of these two parameters: sperm concentration ≤ 15 × 10^6^/ml and/or sperm progressive motility ≤ 32%. We used receiver operating characteristic (ROC) curve analysis to test the accuracy (area under the curve, AUC) with 95% confidence interval, the sensitivity and the specificity in predicting oocyte maturity and achievement of fertilization. All statistical tests were 2-sided, and P values of ≤ 0.05 were considered statistically significant.

### Ethical approval

The experimental protocol has been approved by the internal ethical committee of Demetra ART Center of Florence (Italy). All recruited couples were informed of the aim of the research and signed an informed consent for participating in the prospective study, allowing the collection of follicular fluids (usually discarded after oocyte retrieval during ART procedure) and accepting the transfer of a single blastocyst. The research was conducted in accordance with the Declaration of Helsinki.

## Results

### Baseline characteristics of the two groups of couples

Baseline characteristics and reproductive outcomes in the two groups of ovarian stimulation are reported in Table 1. The two groups differed significantly in the age of the women, which was higher in the FSH + LH group, and in the number of high-quality blastocysts which was higher in the FSH group. Pregnancy rate was insignificantly higher in the FSH group.

### FF steroid levels, inter- and intra-subject variability and correlations between hormone levels in the two stimulation protocols

Median FF steroid levels in women undergoing the two stimulation protocols are reported in Table 2. P, 17-OH-P, E2 and E1 were detectable in all the 316 analyzed FF, whereas A and T levels were detectable respectively in 300 (95%) and 151 (47.8%) follicles. Progestins (P and 17-OH-P) were the most abundant FF steroids in both groups. Among androgens, A levels were higher than T levels in both groups. Levels of 17-OH-P, A, E2 and E1 were significantly higher in FF from women undergoing FSH + LH stimulation than in women undergoing FSH alone whereas no differences were observed for P and T levels (Table 2).

**Table 2:** Median values [IQR] of Progesterone (P), 17α-Hydroxyprogesterone (17-OH-P), Androstenedione (A), Testosterone (T), Estradiol (E2) and Estrone (E1) FF levels, measured by HPLC–MS/MS, in the two groups of ovarian stimulation (FSH and FSH + LH).

Steroid hormone	FSH group (ng/ml)	FSH + LH group (ng/ml)	p
P	6263.7 [2964.4–10137.8] n = 111	6936.7 [3562.4–10989.5] n = 205	0.29
17-OH-P	576.1 [360.8–877.4] n = 111	898.7 [496.5–1394.4] n = 205	**< 0.0001**^**§**^
A	2.8 [1.0–10.0] n = 108	5.3 [2.1–13.7] n = 192	**0.002**
T	0.8 [0.3–0.9] n = 69	0.7 [0.3–0.92] n = 82	0.71
E2	192.2 [127.3–304.9] n = 111	347.2 [219.1–515.7] n = 205	**< 0.0001**^**§**^
E1	14.2 [9.0–22.8] n = 111	20.6 [13.2–34.4] n = 205	**< 0.0001**^**§**^

p column indicates the statistical significance between the two groups of ovarian stimulation. In bold statistically significant differences.

^**§**^Statistical significance was maintained after adjustment for female age.

Inter-subject variability (median value of CVs) of steroid levels of the analyzed FF in the two groups is reported in Fig. 2A. A levels demonstrated the highest inter-subject coefficients of variation in both groups (Fig. 2A). Intra-subject P levels of all analyzed FF, as an example of FF hormone variation in each of the 41 women, is shown in Fig. 2B. Intra-subject variability was similar in the two groups (Fig. 2B). Similar results were observed for the other hormones (not shown).

Table 3 reports correlations between FF steroid levels in the two study groups. In both groups we found the following significantly positive correlations: P with 17-OH-P and E2; 17-OH-P with E2 and E1; A with T, E2 and E1; E2 with E1. In the FSH group we found a positive correlation between T and E1 and negative correlations between P and A and P and T.

**Table 3:** Correlation (reported as Spearman's rank correlation coefficient) among FF steroid hormone levels in the two groups of ovarian stimulation (FSH and FSH + LH).

	P	17-OH-P	A	T	E2	E1
**FSH group**						
P (n = 111)	1	0.74**	− 0.34**	− 0.38*	0.41**	− 0.09
17-OH-P (n = 111)		1	0.02	− 0.02	0.77**	0.31*
A (n = 108)			1	0.65**	0.19*	0.45**
T (n = 69)				1	0.08	0.37*
E2 (n = 111)					1	0.54**
E1 (n = 111)						1
**FSH + LH group**						
P (n = 205)	1	0.87**	− 0.09	− 0.10	0.69**	0.25**
17-OH-P (n = 205)		1	0.12	0.02	0.86**	0.43**
A (n = 192)			1	0.46**	0.21*	0.24*
T (n = 82)				1	0.07	0.05
E2 (n = 205)					1	0.65**
E1 (n = 205)						1

*p < 0.05, **p < 0.001. In bold statistically significant differences.

### Relationship between FF steroid levels and oocyte maturity

Median FF steroid levels in Metaphase II (MII) and immature oocytes in the two groups of subjects are reported in Table 4. In the FSH group, none of the hormones was significantly different between mature and immature oocytes (Table 4). In the FSH + LH group, P, 17-OH-P and E2 levels were significantly higher in MII oocytes (also after adjustment for female age), whereas no differences were observed for the other hormones (Table 4). To understand whether steroid FF levels can distinguish MII oocytes from immature ones, ROC analysis was used as a binary classifier system. P, 17-OH-P and E2 FF levels were able to distinguish MII oocytes with an accuracy of 66.6% (CI 95% 0.575–0.757), 67.0%(CI 95% 0.586–0.755) and 66.0% (CI 95% 0.573–0.746) respectively (p < 0.001 for all).

**Table 4:** Median values [IQR] of Progesterone (P), 17α-Hydroxyprogesterone (17-OH-P), Androstenedione (A), Testosterone (T), Estradiol (E2) and Estrone (E1) levels in FF in the two groups based on oocyte maturity: MII (mature oocytes) and immature oocytes (MI and VG).

Steroid hormone	FSH group			FSH + LH group			
	MII oocytes (ng/ml)	Immature oocytes (ng/ml)	p	MII oocytes (ng/ml)		Immature oocytes (ng/ml)	p
P	6415.3 [3065.3–10123.2] n = 96	4430.0 [2497.8–12382.4] n = 15	0.67	7352.7 [4252.2–11378.2] n = 163		4,270.2 [2466.4–8064.9] n = 42	**0.001**^**§**^
17-OH-P	565.5 [361.2–877.8] n = 96	712.9 [356.8–782.6] n = 15	0.82	991.2 [596.2–1620.4] n = 163		571.4 [352.6–1072.3] n = 42	**0.001**^***§***^
A	2.8 [1.1–9.9] n = 93	4.4 [1.0–10.7] n = 15	0.65	5.2 [1.9–14.5] n = 151		6.7 [2.4–12.7] n = 41	0.43
T	0.8 [0.3–0.9] n = 61	0.8 [0.5–1.0] n = 8	0.38	0.6 [0.3–0.9] n = 66		0.7 [0.4–1.2] n = 16	0.64
E2	190.7 [121.5–308.7] n = 96	246.5 [140.1–305.0] n = 15	0.38	396.0 [238.5–551.4] n = 163		245.9 [165.3–385.6] n = 42	**0.001**^***§***^
E1	13.8 [8.7–22.9] n = 96	14.3 [11.2–20.9] n = 15	0.18	20.7 [13.6–34.4] n = 163		18.3 [11.2–30.2] n = 42	0.35

Data were distinguished between the two groups of ovarian stimulation (FSH and FSH + LH).

p columns indicate the statistical significance between the two groups of mature and immature oocytes. In bold statistically significant differences.

^§^Statistical significance was maintained after adjustment for female age.

**Figure 3:**
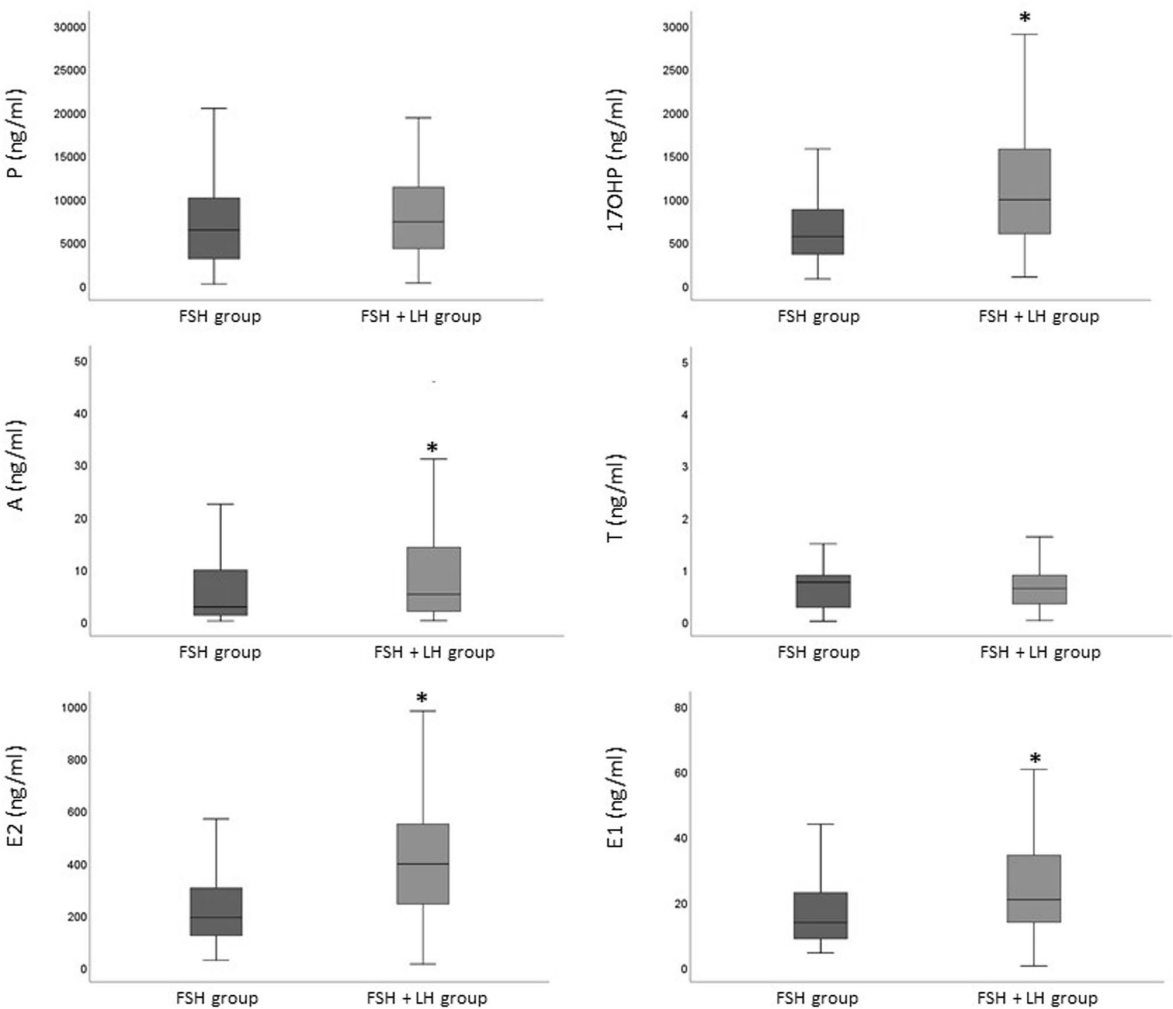
Box plots of median (IQR) FF levels of Progesterone (P, upper left panel), 17α-Hydroxyprogesterone (17-OH-P, upper rightpanel), Androstenedione (A, middle left panel), Testosterone (T, middle right panel), Estradiol (E2, lowerleft panel) and Estrone (E1, lower right panel) levels in mature (MII) oocytes, in the two groups of ovarian stimulation. *p < 0.05 vs FSH group.

In mature oocytes, FF levels of 17-OH-P, A, E2 and E1 were significantly higher in FSH + LH group than the FSH group (Fig. 3) whereas no differences were observed for immature oocytes (not shown).

### Relation between FF steroid levels and IVF outcomes

**Figure 4:**
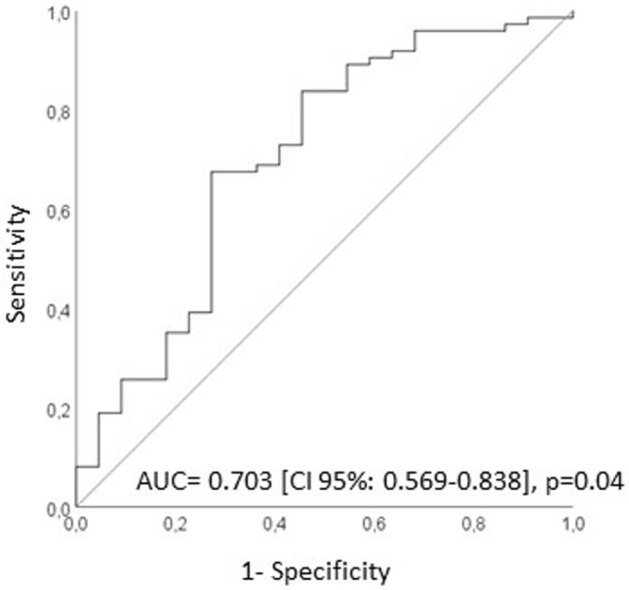
ROC curve of the ability of FF Progesterone (P) levels to predict the attainment of fertilization in FSH group. *AUC* area under the curve, *CI* confidence interval.

In the FSH group, median levels of P were higher in follicles of oocytes achieving fertilization (also after adjustment for female age and male factor) than in non-fertilized oocytes, whereas no significant differences were observed for the other hormones (Table 5). ROC analysis demonstrated that P levels in FF are able to distinguish fertilized oocyte with an accuracy of 70.3% (CI 95% 0.569–0.838, p < 0.05, Fig. 4). Logistic regression analysis showed that when FF P levels were ≥ 5631.584 ng/ml, the odds ratio to obtain correct fertilization was 4.2 (CI 95% 1.38–12.95, p = 0.01).

**Table 5:** Median values [IQR] of Progesterone (P), 17α-Hydroxyprogesterone (17-OH-P), Androstenedione (A), Testosterone (T), Estradiol (E2) and Estrone (E1) levels in FF in the two groups based on fertilization: fertilized and non-fertilized oocytes.

Steroid hormone	FSH group			FSH + LH group		
	Fertilized oocytes (ng/ml)	Non-fertilized oocytes (ng/ml)	p	Fertilized oocytes (ng/ml)	Non-fertilized oocytes (ng/ml)	p
P	6918.3 [4040.7–10972.8] n = 74	3000.7 [1121.4–8095.8] n = 22	**0.004**^**§**^	8292.0 [4840.9–11606.1] n = 119	6376.6 [3647.8–10113.2] n = 44	0.09
17-OH-P	597.9 [373.6–919.1] n = 74	464.4 [226.2–802.6] n = 22	0.11	1077.6 [663.7–1694.6] n = 119	811.9 [362.3–1385.7] n = 44	**0.04**
A	2.3 [0.8–8.1] n = 71	4.0 [1.3–14.2] n = 22	0.23	5.3 [1.9–14.2] n = 113	4.3 [1.9–15.2] n = 38	0.75
T	0.8 [0.3–0.9] n = 48	0.8 [0.3–1.0] n = 13	0.75	0.7 [0.3–0.9] n = 48	0.6 [0.3–0.8] n = 18	0.65
E2	188.1 [120.2–298.7] n = 74	199.2 [126.3–326.1] n = 22	0.66	396.9 [253.2–595.9] n = 119	373.4 [134.8–497.7] n = 44	0.12
E1	12.1 [7.9–22.8] n = 74	14.8 [10.7–23.8] n = 22	0.33	20.7 [13.6–34.4] n = 119	20.2 [12.5–34.9] n = 44	0.57

Data were distinguished between the two groups of ovarian stimulation (FSH and FSH + LH).

p columns indicate the statistical significance between the groups of fertilized and non-fertilized oocytes. In bold statistically significant differences.

^§^Statistical significance was maintained after adjustment for female age and male factor.

In the FSH + LH group, 17-OH-P levels were higher in fertilized oocytes, although significance was lost after adjustment for confounders (Table 5).

Next, we evaluated the relationship between FF steroid levels and the quality of the corresponding blastocysts. Blastocysts were classified in high (HQ), intermediate (IQ) and low (LQ) quality as indicated in the Materials and Methods section. No significant differences were observed in the FF steroid levels in the three embryo categories in both groups of ovarian stimulation (results not shown). Similarly, no significant differences were found in FF steroid levels of oocytes resulting in clinical pregnancy in both groups (results not shown).

## Discussion

To our knowledge, this is the largest study collecting single FF of women undergoing IVF and tracking the fate of the corresponding oocytes. Since steroid FF levels vary substantially among different follicles collected from the same woman [Ref.^34^ and present study], individually tracking the fate of all retrieved oocytes and evaluating steroids in each FF appears to be the best strategy to evaluate the relationship between ovarian stimulation protocol and FF steroid composition as well as between FF steroid levels and oocyte maturity and IVF outcomes.

### FF steroid composition is affected by the ovarian stimulation protocol

Our study demonstrates that providing LH activity from the start of ovarian stimulation to women with a previous unexpected deficient or suboptimal ovarian response^7,8,29^ results in significant differences in the steroid composition of FF, which might have contributed to improving the ovarian response. In particular, our results suggest that, with LH supplementation, the follicular steroidogenic activity leads to an FF steroidal pattern resembling the one occurring during physiological follicle maturation^35^. Indeed, the high levels of progestins and estrogens detected in the FSH + LH group suggest an increased production of these hormones during oocyte maturation, similar to what normally occurs in the physiological condition^35^.

Follicular steroidogenesis is promoted by the coordinated actions of FSH and LH. FSH increases CYP19A (aromatase) and LH receptor expressions in granulosa cells, increasing their sensitivity to LH. LH enhances the synthesis of androgens in the theca cells and promotes the expression of CYP11A1 (P450 cholesterol side chain cleavage) and CYP19A in granulosa cells^36^. The action of gonadotrophins on the enzyme complex CYP17 has been studied less. In humans, CYP17 is able to hydroxylase pregnenolone to 17-hydroxypregnenolone and P to 17-OH-P with the same efficiency, but its 17–20 lyase activity is exerted only on 17-hydroxypregnenolone, which is converted to DHEA. In fact, androgens, in physiological conditions, are produced preferentially through the ∆5 pathway^37^. It is well known that ovarian stimulation in ARTs leads to supraphysiological steroid FF concentrations^3,38^. Our results, showing that in the FSH + LH protocol the production of most steroids increased, appear in agreement with the described upregulating effect of LH on CYP11A1. Considering that P derives from the conversion of pregnenolone through the action of 3β-HSD (the delta4 pathway), the activity of this enzyme does not appear to be enhanced by the addition of LH, as P levels are similar in the two groups. Conversely, theca cell stimulation with LH appears to increase the activity of CYP17 enzyme, which converts P to 17-OH-P. The increased activity of CYP17, on the other hand, could be responsible also for the increase of A from the delta-5 pathway, although the activation of the delta-4 pathway cannot be excluded. This hypothesis is supported by the correlation between 17-OH-P and estrogens in both groups. The increased levels of E1 and E2, but not of T, in the FSH + LH protocol, suggests an increased aromatization of A in granulosa cells. Overall, the modifications of FF steroid levels and composition may explain why, despite being characterized by a significantly higher age and lower blastocyst quality, the percentage of mature oocytes, the fertilization rate and the pregnancy rate of women treated with LH supplementation was not significantly different from that of women treated with FSH monotherapy (Table 1). Thus, in line with the recommendation proposed by Poseidon group^7,8,29^, our study confirms that women with normal ovarian reserve with a history of suboptimal or poor ovarian response might benefit from FSH and LH co-treatment.

Previous studies comparing FF steroid hormone levels in women stimulated with FSH or FSH + LH (both recombinant or using HP-hMG) reported inconsistent results likely due to different study designs and LH sources. The study by Hill and Osteen^10^, evaluating FF steroid levels in follicles larger than 17 mm, found higher levels of E2 and A and lower levels of P in FSH + LH (hMG) treated women. More recently, Barberi et al.^12^ reported higher P but lower E2 levels in FF from follicles of 18–22 mm containing an MII oocyte of FSH + LH (both recombinant) stimulated women. In the large multi-centric randomized study by Smitz et al.^11^, evaluating steroid levels in one FF of follicle ≥ 17 mm from each woman, higher E2, A and T and lower P levels were found in FSH + LH (HP-hMG) stimulated women. All of these studies employed an immunometric method to evaluate steroids in FF. Overall, our study, although with a different design, patient selection and method to evaluate steroids, show similar results to those of Smitz et al.^11^ and Westergaard et al.^5^ regarding E2 and A levels.

### FF steroid levels influence oocyte maturity and IVF outcomes

In agreement with most previous studies^13,39–41^, we found that FF progestin and estrogen levels were higher in MII oocytes at least in the group of women stimulated with the FSH + LH protocol. Conversely, in the FSH group neither progestin nor estrogen levels were significantly different between mature and immature oocytes, likely because of the low number of immature oocytes in this group. The association between progestins and E2 levels and oocyte maturity in the FSH + LH group further corroborate the hypothesis that supplementation with LH leads to a steroid pattern more similar to that seen in late physiological pre-ovulatory follicles that support oocyte maturation (see above^35^). It is well known that FF steroids may influence oocyte differentiation both via genomic and non-genomic action^42^. Whereas genomic effects usually occur at low steroid levels, non-genomic ones occur at much higher concentrations^43^. In particular, rapid, non-genomic effects of P and E2 on maturing oocytes have been observed at µM concentrations and such effects appear to be important in supporting maturation^44–46^. We can speculate that the higher concentrations of E1, E2 and 17-OH-P found in FF of mature oocytes of FSH + LH stimulated women compared to the FSH group (Fig. 3) are necessary to activate the non-genomic pathways to promote oocyte maturation.

We found that progestin levels are higher in FF from those oocytes resulting in correct fertilization, in agreement with a recent meta-analysis^16^. In particular, we have shown that FF P levels can identify oocytes undergoing correct fertilization with fair accuracy and an OR of 4.2 in women stimulated with the FSH protocol. However, whereas in the FSH group P seems to be the main FF steroid involved in the acquisition of the oocyte competence to be fertilized, the role of P in the FSH + LH groups is less clear, as, even if both progestins are higher in fertilized oocytes, full statistical significance was not achieved. We can speculate that the increase of most FF steroids occurring after LH supplementation, modifying the follicular microenvironment in MII oocytes, changes the contribution of single hormones in the development of oocyte competence to be fertilized.

Overall, our results further corroborate the role of progestins in promoting oocyte maturity as well as fertilization competence^13,16,41,42^. Conversely, a role in fertilization competence of other steroid hormones did not emerge from our study.

No differences were found in steroid concentrations among those FF giving rise to high, intermediate or low quality embryos, in agreement with a previous study^18^. Similarly, no differences were found in steroid levels of FF resulting in clinical pregnancy. Embryo development and quality depends on intrinsic characteristics of both gametes, which likely contribute equally. Recently, we demonstrated a relationship between some sperm characteristics and embryo quality^47^. Successful implantation, on the other hand, depends on embryo quality^47–49^, as well as several other factors related to the receiving woman.

Our study has strengths and limitations. As mentioned, tracking the fate of all retrieved individual oocytes in a large number of FF is a strength of our study. Another strength regards the use of the highly reproducible and accurate HPLC–MS/MS method to evaluate steroid levels in FF^23^. Although we recruited quite a large number of women for this type of study, the low absolute number of embryos in the three quality categories may limit the statistical power regarding the role of FF steroids in predicting embryo quality and the subsequent achievement of pregnancy.

In conclusion, our study demonstrates that stimulation of women with FSH + LH protocol results in a different composition of FF showing increased steroid production and a better similarity with the physiological pattern. Such differences might explain why higher ovarian responsiveness was observed in women with previous hypo-responsiveness to ovarian stimulation with FSH protocol^9,29,50^. Employing a correct methodological procedure to evaluate the relationship between FF steroid hormones and ART outcomes, our study reveals that some steroids in single follicles may be helpful in predicting oocyte maturity and attainment of fertilization.

## Supplementary information

Supplementary Tables
