# Supplementary material for: LH supplementation of ovarian stimulation protocols influences follicular fluid steroid composition contributing to the improvement of ovarian response in poor responder women
Source: Sci Rep. 2020 Jul 31;10:12907. doi: 10.1038/s41598-020-69325-z (PMC7395161; doi:10.1038/s41598-020-69325-z)
Supplement: Supplementary file 1 — Supplementary Tables [file 41598_2020_69325_MOESM1_ESM.pdf]

LH SUPPLEMENTATION OF OVARIAN STIMULATION PROTOCOLS INFLUENCES FOLLICULAR FLUID STEROID COMPOSITION CONTRIBUTING TO IMPROVE OVARIAN RESPONSE IN POOR RESPONDER WOMEN

S. Marchiani, L. Tamburrino, F. Benini, M. Pallecchi, C. Bignozzi, A. Conforti, C. Alviggi, L. Vignozzi, G. Danza, S. Pellegrini, E. Baldi

Supplemental Table I. Embryo classification in high quality (HQ), intermediate quality (IQ) and low quality (LQ) according to morphological criteria: the expansion grade scale ranges from 1 (least expanded) to 6 (completely hatched); inner cell mass grading is indicated by A (many cells, tightly packed), B (several cells, loosely packed) and C (very few cells); the trophectoderm is indicated by A (many cells, forming a cohesive layer), B (few cells, forming a loose layer), C (very few large cells).

Embryo classification	Expansion grading	Inner cell mass grading	Trophectoderm grading
High quality	1, 2, 3, 4, 5, 6	A	A
	3, 4, 5, 6	A	B
	3, 4, 5, 6	B	A
	3, 4, 5, 6	B	B
Intermediate quality	1, 2	A	B
	1, 2	B	A
	1, 2	B	B
	3, 4, 5, 6	B	C
	3, 4, 5, 6	C	B
Low quality	1, 2	B	C
	1, 2	C	B
	1, 2, 3, 4, 5, 6	C	C

Supplemental Table II. MRM Parameters for MS/MS Experiments.

**Transition**	**Q1 (m/z)**	**Q3 (m/z)**	**Dwell Time (msec)**	**CE (eV)**
Testosterone Quantifier	289	109	50	30
Testosterone Qualifier	289	97	50	27
**Testosterone-d3**	**292**	**97**	**50**	**27**
Androstendione Quantifier	287	97	50	27
Androstendione Qualifier	287	109	50	30
**Androstenedione-$^{13}C_3$**	**290**	**100**	**50**	**30**
Progesterone Quantifier	315	97	50	25
Progesterone Qualifier	315	109	50	30
**Progesterone-$^{13}C_3$**	**318**	**100**	**50**	**25**
17-OH Prog Quantifier	331	109	50	30
17-OH Prog Qualifier	331	97	50	30
**17-OH Prog-$^{13}C_3$**	**334**	**100**	**50**	**30**
Estradiol Quantifier	271	183	50	-55
Estradiol Qualifier	271	145	50	-55
**Estradiol-d3**	**274**	**145**	**50**	**-50**
Estrone Quantifier	269	183	50	-50
Estrone Qualifier	269	145	50	-50
**Estrone-d4**	**273**	**147**	**50**	**-50**

The transitions for the internal standards are bolded.
